# Reperfusion and Compartment Syndrome After Flexible Ureteroscopy in a Patient with an Iliac Vascular Graft

**DOI:** 10.1089/cren.2016.0108

**Published:** 2016-11-01

**Authors:** Esteban Emiliani, Michele Talso, Edgar Beltrán-Suárez, Steeve Doizi, Olivier Traxer

**Affiliations:** ^1^ Department of Urology, Hôpital Tenon, Université Pierre et Marie Curie Paris VI, Paris, France.; ^2^ Groupe de Recherche Clinique sur la Lithiase Urinaire, GRC n° 20, Sorbonne Universités, Paris VI, France.

**Keywords:** ureteroscopy, lithotomy, position, vascular, complications

## Abstract

***Background:*** Flexible ureteroscopy (fURS) is one of the main treatment options for urolithiasis less than 2 cm. Although fURS has no relative contraindication, some anatomical factors may need to be considered, as not all patients are suitable for the regular lithotomy position (LP). We report the case of a patient with a right iliac vascular graft that after an fURS without intraoperative incidences developed a reperfusion syndrome of the right lower limb.

***Case Presentation:*** A 46-year-old male patient was referred for treatment and follow-up in the cystinuric clinic after being found to have a 3 cm pelvic stone with a Double-J catheter in place after two failed sessions of shockwave lithotripsy. The patient was placed in the LP and a standard ureteroscopy was done with no intraoperative complications. During the first hour in the recovery room, the patient developed severe pain in the right calf muscle stiffness, edema, and increased volume. A postreperfusion and compartment syndrome diagnosis was made with emergency fasciotomy.

***Conclusion:*** To perform fURS, each case must be assessed individually. If a patient with an iliac vascular graft has to undergo fURS, the patient positioning must be modified by keeping the ipsilateral (or both) legs straight to avoid graft complications.

## Introduction and Background

Flexible ureteroscopy (fURS) is one of the main treatment options for urolithiasis less than 2 cm,^1^ providing good outcomes and low complication rates.^2^

There are no relative contraindications for fURS besides anesthesia or active urinary infections,^1^ each case must be evaluated individually to detect possible complications to avoid them.

**FIG. 1.**
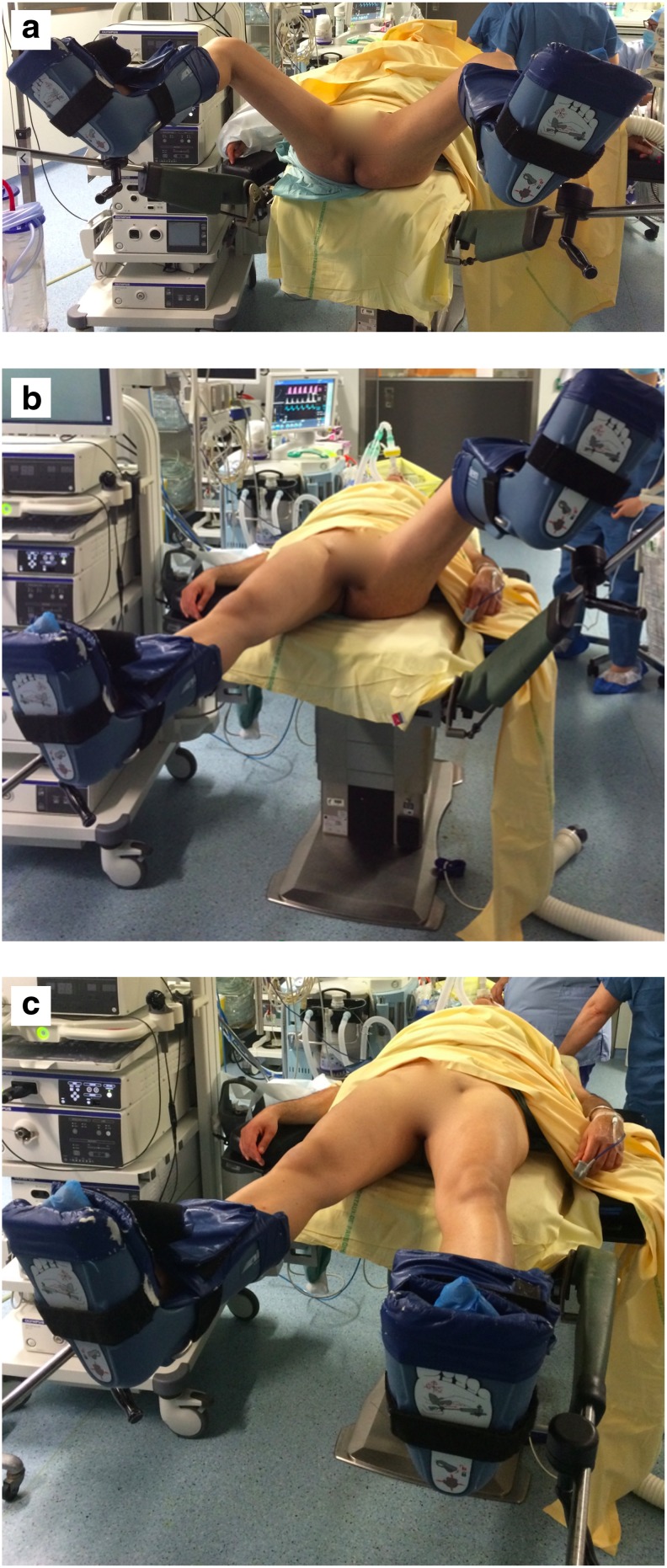
**(a)** Lithotomy position. **(b)** Modified lithotomy position with leg extended on the graft side. **(c)** Dorsal supine.

The majority of patients are placed in the lithotomy position (LP) for a standard fURS^3^ (Fig. 1a), but some anatomical factors may restrict it on some patients.

We report the case of a patient with a right iliac vascular graft that after an fURS session without significant intraoperative events developed a reperfusion syndrome of the right leg because of the LP.

## Case Presentation

A 46-year-old male patient was referred for treatment and follow-up in the cystinuric clinic.

Medical history included type 2 diabetes, hypertension, obesity (body mass index [BMI] 39.5), dyslipidemia, a heavy smoking background (three packs a day for 32 years), and a peripheral arterial disease that led to an internal right iliac artery graft placement 6 years ago. In 1994, cystinuria was diagnosed with a complicated kidney staghorn stone that led to a left nephrectomy.

Since then, multiple fURS sessions were performed.

The patient arrived at our urology department after being found to have a 3 cm pelvic stone with a Double-J catheter placed after two shockwave lithotripsy failed sessions (the last one was discontinued because of his overweight). At that time, his glomerular filtration rate was 59 mL/(min ·1.73 m^2^).

After treatment options were explained, an fURS session was planned and performed. The patient was placed in the LP as standard procedure. After 2 hours, the stone was completely dusted and a Double-J catheter was left in place. There were no intraoperative complications noted.

During the first hour in the recovery room, the patient developed severe pain in the right calf. Physical examination by a vascular surgeon showed muscle stiffness, edema, and increased volume; the patient did not tolerate external compression and had no sensory loss of or motor response failure.

Doppler ultrasound examination showed femoral, popliteal, and posterior tibial pulses. The vascular graft was permeable and the popliteal and posterior tibial arteries had adequate flow rates.

The patient was found to have a postreperfusion and compartment syndrome after suffering from an ischemic period because of prosthesis bending and collapsing in the LP. An urgent fasciotomy was performed to release the lower limb pressure. A double incision technique was done with two 15 cm incisions in the anterolateral and posteromedial areas of the calf muscle to decompress the anterior and lateral compartments and the superficial posterior and deep posterior compartments, respectively.

The patient was discharged 5 days later after delayed primary skin closure with no further incidences.

Seven weeks later the surgical wounds were healing properly, during which time the patient underwent physiotherapy, recovering completely. The Double-J catheter was removed and a complete metabolic evaluation was initiated for his cystinuria being stone free.

## Discussion

One of the main advantages of fURS is the relative small complication rate. The CROES study group reported an overall 3.5% rate in their 11,885 patients series.^2^ Although most of the complications in fURS are nonlife threatening (98% Clavien–Dindo I–III^4^), severe complications have also been reported and endourologists must take them into consideration.^5^

Low complication rates are one of the reasons fURS has increased, its feasibility and safety have been proven for complex patients such as pregnant or obese patients (as in the current case) or patients with bleeding diathesis.^6^ Furthermore, there are no specific contraindications (except anesthesia or active urinary infections).^1^ Treatment must be tailored and each patient must be assessed individually to keep the procedure safe.

In our case, the LP bended the patient's graft and caused a collapse, causing a 2-hour ischemia time and developing a postreperfusion syndrome afterward. This can be classified as a Clavien–Dindo IIIb complication, needing urgent surgery and anesthesia. This condition requires quick diagnosis and treatment as untreated reperfusion syndromes within the first 6 hours can lead to functional deterioration or irreversible rhabdomyolysis and limb loss.^7^ Normal nerve conduction can tolerate 2 hours of ischemia and muscles can tolerate up to 5 hours.^6^

Leff and Shapiro first described the reperfusion syndrome after LP in 1979.^8^ Later, it has been reported in patients after radical cystectomy, prostatectomy, and urethroplasty in Trendelemburg or LP in prolonged surgeries up to 11 hours.^9^ Most of the cases reported in the literature are patients without grafts and mainly after prolonged colorectal surgeries.^8–13^ In patients with vascular implants, only aortic endograft thrombosis has been reported again after colorectal surgery.^10,11^

The risks factors for reperfusion syndrome are long surgeries (>5 hours),^12–14^ LP combined with Trendelemburg position,^15^ and external compression (i.e., stockings or abdominal or wound retractors),^16^ although the risk factors are not usually seen during fURS. The physiopathology includes decreased perfusion when elevating the limbs above the heart level, intraoperative low blood pressure, or arterial or venous insufficiency.^8,13^

Finally we note that even during short surgeries, the LP may cause complications in patients with iliac vascular grafts. In patients with vascular grafts, hip replacement or severe hip ankylosis^17^ fURS can be performed modifying the patient's position to avoid the graft's occlusion.

They can be operated on a complete supine position (Fig. 1c), with the surgeon placed laterally or keeping the graft's ipsilateral leg horizontal (Fig. 1b). A flexible cystoscopy can be initially performed as well as a standard fURS. This simple but substantial modification in technique will prevent vascular graft complications.

Furthermore, depending on the case and stone size, a percutaneous nephrolithotomy can be performed as standard alternative. The patient could be placed either in complete supine (Valdivia) position^18^ or in prone position with a modified split leg position^19^; both for endoscopic combined approach. Under these circumstances, both lower limbs will remain horizontally, avoiding this type of injury.

## Conclusion

To perform fURS, each case must be evaluated individually. If a patient with an iliac vascular graft undergoes fURS, the positioning must be modified by keeping the ipsilateral (or both) legs horizontally to avoid complications with the graft.

## Abbreviations Used

fURS: flexible ureteroscopy
LP: lithotomy position
